# 1,4-Bis(benzimidazol-2-yl)benzene dimethyl­formamide disolvate

**DOI:** 10.1107/S1600536809004759

**Published:** 2009-02-13

**Authors:** De-Hong Wu, Ling Hu

**Affiliations:** a Ordered Matter Science Research Center, College of Chemistry and Chemical Engineering, Southeast University, Nanjing 210096, People’s Republic of China

## Abstract

The aromatic mol­ecule of the title compound, C_20_H_14_N_4_·2C_3_H_7_NO, occupies a special position on an inversion center. The benzimidazole unit (planar to within 0.008 Å) forms a dihedral angle of 9.1 (2)° with the central benzene ring. The benzimidazole H atom participates in a hydrogen bond with the dimethyl­formamide solvent molecule, thus giving rise to the title 1:2 aggregate. These aggregates are further linked in the crystal structure by aromatic π–π stacking inter­actions [centroid–centroid distance = 6.356 (2) Å].

## Related literature

For background literature concerning benzimidazole compounds, see: Zarrinmayeh *et al.* (1998 ▶); Gallagher *et al.* (2001 ▶); Howarth & Hanlon (2001 ▶). For the unsolvated structure, see: Bei *et al.* (2000 ▶); Dudd *et al.* (2003 ▶).
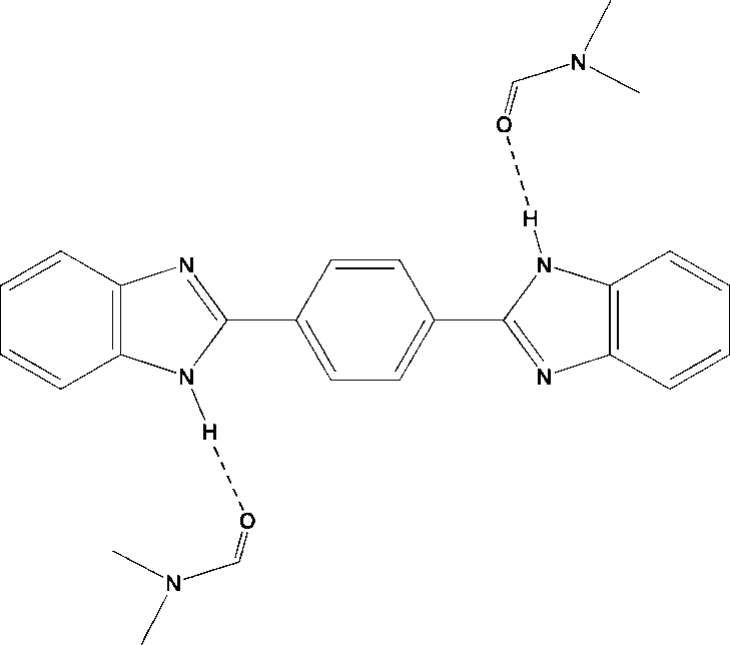


## Experimental

### 

#### Crystal data

C_20_H_14_N_4_·2C_3_H_7_NO
*M* *_r_* = 456.54
Monoclinic, 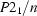

*a* = 6.3556 (13) Å
*b* = 20.931 (2) Å
*c* = 9.0097 (18) Å
β = 98.26 (2)°
*V* = 1186.1 (4) Å^3^
*Z* = 2
Mo *K*α radiation
μ = 0.08 mm^−1^
*T* = 291 K
0.32 × 0.26 × 0.24 mm

#### Data collection

Rigaku Mercury2 diffractometer
Absorption correction: multi-scan (*CrystalClear*; Rigaku, 2005 ▶) *T*_min_ = 0.970, *T*_max_ = 0.990
12310 measured reflections
2723 independent reflections
1718 reflections with *I* > 2σ(*I*)
*R*_int_ = 0.054

#### Refinement

*R*[*F* ^2^ > 2σ(*F* ^2^)] = 0.057
*wR*(*F* ^2^) = 0.158
*S* = 1.00
2723 reflections
154 parameters
H-atom parameters constrained
Δρ_max_ = 0.24 e Å^−3^
Δρ_min_ = −0.21 e Å^−3^

### 

Data collection: *CrystalClear* (Rigaku, 2005 ▶); cell refinement: *CrystalClear*; data reduction: *CrystalClear*; program(s) used to solve structure: *SHELXS97* (Sheldrick, 2008 ▶); program(s) used to refine structure: *SHELXL97* (Sheldrick, 2008 ▶); molecular graphics: *SHELXTL* (Sheldrick, 2008 ▶); software used to prepare material for publication: *SHELXTL*.

## Supplementary Material

Crystal structure: contains datablocks I, global. DOI: 10.1107/S1600536809004759/ya2086sup1.cif

Structure factors: contains datablocks I. DOI: 10.1107/S1600536809004759/ya2086Isup2.hkl

Additional supplementary materials:  crystallographic information; 3D view; checkCIF report

## Figures and Tables

**Table 1:** Hydrogen-bond geometry (Å, °)

*D*—H⋯*A*	*D*—H	H⋯*A*	*D*⋯*A*	*D*—H⋯*A*
N2—H2*A*⋯O1^i^	0.86	1.95	2.787 (3)	165

Symmetry code: (i) 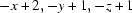.

**Fig. 1.**
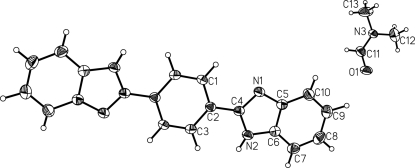
The structure of 1,4-bis(2-benzimidazolyl)benzene and dimethylformamide molecules in the crystal of the title compound, showing the atomic numbering scheme and 30% probability displacement ellipsoids. Unlabelled atoms are related to the labelled atoms by the (1 - x, -y, 1 - z) symmetry transformation.

## supplementary crystallographic information

### Comment

Benzimidazole systems continue to attract considerable attention in chemical synthesis, structural science and applied medicinal research (Zarrinmayeh *et al.*, 1998; Gallagher *et al.*, 2001; Howarth & Hanlon, 2001). Here we report the crystal structure of the title compound, 1,4-bis(2-benzimidazolyl)benzene bis(dimethylformamide) solvate.

The 1,4-bis(2-benzimidazolyl)benzene molecule occupies a special position on the inversion center, and benzimidazole moiety (planar within 0.0078 Å) forms dihedral angle of 9.1 (2)° with the plane of the central benzene ring (Fig. 1). This shows that 1,4-(2-benzimidazolyl)benzene molecule in the structure of the title compound deviates from planarity to a much lesser degree than in the unsolvated structure, wherein the corresponding dihedral angle is equal to 31.0° (Bei *et al.*, 2000; Dudd *et al.*, 2003).

The only `active' hydrogen atom H2 participates in the H-bond with the carbonyl atom of the dimethylformamide molecule (H2···O1 1.95 Å, N2—H2···O1 165.1°) thus giving rise to the 1,4-bis(2-benzimidazolyl)benzene:DMFA (1:2) complexes, which are further linked in crystal through the π—π stacking interactions.

### Experimental

The title compound was synthesized by refluxing terephthalaldehyde (0.53 g, 4 mmol) and benzene-1,2-diamine (0.86 g, 8 mmol) in absolute methanol (50 ml) for 8 h. After cooling to room temperature, the yellow solid thus formed was isolated and dried under vacuum (1.13 g, yield 80 %). Single crystals suitable for X-ray structure analysis were obtained by the slow evaporation of a dimethylformamide solution in air.

### Refinement

H atoms were placed in calculated positions (N—H = 0.86 Å; C—H = 0.93 Å and 0.96 Å for C*sp*^2^ and C*sp*^3^ atoms, respectively), assigned fixed *U*_iso_ values [*U*_iso_ = 1.2*U*eq(C*sp*^2^/N) and 1.5*U*eq(C*sp*^3^)] and allowed to ride.

### Crystal data

**Table:**

C_20_H_14_N_4_·2C_3_H_7_NO	*F*(000) = 484
*M**_r_* = 456.54	*D*_x_ = 1.278 Mg m^−^^3^
Monoclinic, *P*2_1_/*n*	Mo *K*α radiation, λ = 0.71073 Å
Hall symbol: -P 2yn	Cell parameters from 9216 reflections
*a* = 6.3556 (13) Å	θ = 3.0–27.7°
*b* = 20.931 (2) Å	µ = 0.08 mm^−^^1^
*c* = 9.0097 (18) Å	*T* = 291 K
β = 98.26 (2)°	Block, yellow
*V* = 1186.1 (4) Å^3^	0.32 × 0.26 × 0.24 mm
*Z* = 2	

### Data collection

**Table:**

Rigaku Mercury2 diffractometer	2723 independent reflections
Radiation source: fine-focus sealed tube	1718 reflections with *I* > 2σ(*I*)
graphite	*R*_int_ = 0.054
Detector resolution: 13.6612 pixels mm^-1^	θ_max_ = 27.5°, θ_min_ = 3.0°
CCD profile fitting scans	*h* = −8→8
Absorption correction: multi-scan (*CrystalClear*; Rigaku, 2005)	*k* = −27→27
*T*_min_ = 0.970, *T*_max_ = 0.990	*l* = −11→11
12310 measured reflections	

### Refinement

**Table:**

Refinement on *F*^2^	Primary atom site location: structure-invariant direct methods
Least-squares matrix: full	Secondary atom site location: difference Fourier map
*R*[*F*^2^ > 2σ(*F*^2^)] = 0.057	Hydrogen site location: inferred from neighbouring sites
*wR*(*F*^2^) = 0.158	H-atom parameters constrained
*S* = 1.00	*w* = 1/[σ^2^(*F*_o_^2^) + (0.0615*P*)^2^ + 0.4757*P*] where *P* = (*F*_o_^2^ + 2*F*_c_^2^)/3
2723 reflections	(Δ/σ)_max_ < 0.001
154 parameters	Δρ_max_ = 0.24 e Å^−^^3^
0 restraints	Δρ_min_ = −0.21 e Å^−^^3^

### Special details

**Table:**

Geometry. All esds (except the esd in the dihedral angle between two l.s. planes) are estimated using the full covariance matrix. The cell esds are taken into account individually in the estimation of esds in distances, angles and torsion angles; correlations between esds in cell parameters are only used when they are defined by crystal symmetry. An approximate (isotropic) treatment of cell esds is used for estimating esds involving l.s. planes.
Refinement. Refinement of F^2^ against ALL reflections. The weighted R-factor wR and goodness of fit S are based on F^2^, conventional R-factors R are based on F, with F set to zero for negative F^2^. The threshold expression of F^2^ > 2sigma(F^2^) is used only for calculating R-factors(gt) etc. and is not relevant to the choice of reflections for refinement. R-factors based on F^2^ are statistically about twice as large as those based on F, and R- factors based on ALL data will be even larger.

### Fractional atomic coordinates and isotropic or equivalent isotropic displacement parameters (Å^2^)

**Table:**

	*x*	*y*	*z*	*U*_iso_*/*U*_eq_	
C1	0.4424 (3)	0.03145 (10)	0.6222 (2)	0.0442 (5)	
H1A	0.4027	0.0527	0.7045	0.053*	
C2	0.6334 (3)	0.04722 (9)	0.5722 (2)	0.0394 (5)	
C3	0.6877 (3)	0.01491 (10)	0.4482 (2)	0.0454 (5)	
H3A	0.8140	0.0249	0.4126	0.054*	
C4	0.7705 (3)	0.09548 (9)	0.6530 (2)	0.0396 (5)	
C5	0.9076 (3)	0.16201 (10)	0.8206 (2)	0.0431 (5)	
C6	1.0397 (3)	0.16157 (9)	0.7099 (2)	0.0425 (5)	
C7	1.2235 (4)	0.19770 (11)	0.7212 (3)	0.0563 (6)	
H7A	1.3119	0.1966	0.6474	0.068*	
C8	1.2694 (4)	0.23526 (12)	0.8464 (3)	0.0655 (7)	
H8A	1.3914	0.2604	0.8575	0.079*	
C9	1.1379 (4)	0.23673 (12)	0.9574 (3)	0.0638 (7)	
H9A	1.1734	0.2630	1.0404	0.077*	
C10	0.9577 (4)	0.20035 (11)	0.9471 (3)	0.0578 (6)	
H10A	0.8713	0.2012	1.0221	0.069*	
C11	0.7511 (4)	0.87860 (14)	0.7174 (3)	0.0663 (7)	
H11A	0.6668	0.8486	0.6607	0.080*	
C12	0.7988 (5)	0.95068 (16)	0.9240 (3)	0.0905 (10)	
H12A	0.9305	0.9584	0.8865	0.136*	
H12B	0.8277	0.9357	1.0255	0.136*	
H12C	0.7187	0.9897	0.9210	0.136*	
C13	0.4746 (5)	0.88579 (18)	0.8728 (4)	0.0984 (11)	
H13A	0.4093	0.8540	0.8042	0.148*	
H13B	0.3850	0.9229	0.8681	0.148*	
H13C	0.4937	0.8689	0.9728	0.148*	
N1	0.7398 (3)	0.12012 (9)	0.78322 (19)	0.0470 (5)	
N2	0.9489 (3)	0.11884 (8)	0.60413 (19)	0.0442 (4)	
H2A	0.9957	0.1087	0.5223	0.053*	
N3	0.6781 (3)	0.90317 (9)	0.8324 (2)	0.0526 (5)	
O1	0.9230 (3)	0.89199 (11)	0.6780 (2)	0.0845 (6)	

### Atomic displacement parameters (Å^2^)

**Table:**

	*U*^11^	*U*^22^	*U*^33^	*U*^12^	*U*^13^	*U*^23^
C1	0.0431 (11)	0.0534 (13)	0.0382 (11)	0.0016 (10)	0.0135 (9)	−0.0061 (9)
C2	0.0390 (11)	0.0442 (11)	0.0355 (10)	0.0044 (9)	0.0071 (8)	0.0028 (8)
C3	0.0397 (11)	0.0537 (12)	0.0451 (12)	−0.0003 (10)	0.0144 (9)	−0.0011 (10)
C4	0.0386 (11)	0.0425 (11)	0.0385 (10)	0.0033 (9)	0.0088 (9)	0.0043 (9)
C5	0.0431 (11)	0.0414 (11)	0.0457 (12)	0.0015 (9)	0.0088 (9)	0.0011 (9)
C6	0.0441 (11)	0.0398 (11)	0.0440 (11)	0.0015 (9)	0.0080 (9)	0.0061 (9)
C7	0.0494 (13)	0.0570 (14)	0.0655 (15)	−0.0081 (11)	0.0183 (11)	0.0020 (12)
C8	0.0543 (15)	0.0584 (15)	0.0827 (19)	−0.0146 (12)	0.0066 (13)	−0.0018 (13)
C9	0.0672 (16)	0.0582 (15)	0.0649 (16)	−0.0106 (13)	0.0052 (13)	−0.0125 (12)
C10	0.0635 (15)	0.0580 (14)	0.0537 (14)	−0.0070 (12)	0.0146 (12)	−0.0107 (11)
C11	0.0706 (17)	0.0797 (18)	0.0501 (14)	0.0029 (14)	0.0129 (13)	−0.0001 (13)
C12	0.112 (3)	0.085 (2)	0.0701 (19)	0.0047 (19)	−0.0020 (18)	−0.0122 (16)
C13	0.078 (2)	0.126 (3)	0.101 (2)	−0.001 (2)	0.0443 (19)	0.023 (2)
N1	0.0467 (10)	0.0530 (10)	0.0439 (10)	−0.0042 (8)	0.0158 (8)	−0.0052 (8)
N2	0.0454 (10)	0.0488 (10)	0.0414 (9)	−0.0018 (8)	0.0166 (8)	−0.0012 (8)
N3	0.0540 (11)	0.0625 (12)	0.0438 (10)	0.0004 (9)	0.0153 (9)	0.0007 (9)
O1	0.0695 (12)	0.1293 (18)	0.0613 (12)	0.0077 (12)	0.0318 (10)	0.0092 (11)

### Geometric parameters (Å, °)

**Table:**

C1—C3^i^	1.370 (3)	C8—H8A	0.9300
C1—C2	1.394 (3)	C9—C10	1.367 (3)
C1—H1A	0.9300	C9—H9A	0.9300
C2—C3	1.391 (3)	C10—H10A	0.9300
C2—C4	1.459 (3)	C11—O1	1.229 (3)
C3—C1^i^	1.370 (3)	C11—N3	1.300 (3)
C3—H3A	0.9300	C11—H11A	0.9300
C4—N1	1.322 (2)	C12—N3	1.441 (3)
C4—N2	1.364 (2)	C12—H12A	0.9600
C5—N1	1.384 (3)	C12—H12B	0.9600
C5—C10	1.393 (3)	C12—H12C	0.9600
C5—C6	1.393 (3)	C13—N3	1.440 (3)
C6—N2	1.372 (3)	C13—H13A	0.9600
C6—C7	1.383 (3)	C13—H13B	0.9600
C7—C8	1.372 (4)	C13—H13C	0.9600
C7—H7A	0.9300	N2—H2A	0.8600
C8—C9	1.393 (4)		
			
C3^i^—C1—C2	120.91 (18)	C8—C9—H9A	119.3
C3^i^—C1—H1A	119.5	C9—C10—C5	117.9 (2)
C2—C1—H1A	119.5	C9—C10—H10A	121.1
C3—C2—C1	118.20 (19)	C5—C10—H10A	121.1
C3—C2—C4	122.55 (18)	O1—C11—N3	125.0 (3)
C1—C2—C4	119.23 (17)	O1—C11—H11A	117.5
C1^i^—C3—C2	120.89 (19)	N3—C11—H11A	117.5
C1^i^—C3—H3A	119.6	N3—C12—H12A	109.5
C2—C3—H3A	119.6	N3—C12—H12B	109.5
N1—C4—N2	112.48 (18)	H12A—C12—H12B	109.5
N1—C4—C2	124.01 (18)	N3—C12—H12C	109.5
N2—C4—C2	123.48 (17)	H12A—C12—H12C	109.5
N1—C5—C10	129.9 (2)	H12B—C12—H12C	109.5
N1—C5—C6	110.11 (18)	N3—C13—H13A	109.5
C10—C5—C6	120.0 (2)	N3—C13—H13B	109.5
N2—C6—C7	132.4 (2)	H13A—C13—H13B	109.5
N2—C6—C5	105.38 (17)	N3—C13—H13C	109.5
C7—C6—C5	122.2 (2)	H13A—C13—H13C	109.5
C8—C7—C6	116.9 (2)	H13B—C13—H13C	109.5
C8—C7—H7A	121.6	C4—N1—C5	104.84 (16)
C6—C7—H7A	121.6	C4—N2—C6	107.19 (16)
C7—C8—C9	121.6 (2)	C4—N2—H2A	126.4
C7—C8—H8A	119.2	C6—N2—H2A	126.4
C9—C8—H8A	119.2	C11—N3—C13	122.5 (3)
C10—C9—C8	121.5 (2)	C11—N3—C12	120.5 (2)
C10—C9—H9A	119.3	C13—N3—C12	117.0 (2)

Symmetry codes: (i) −*x*+1, −*y*, −*z*+1.

### Hydrogen-bond geometry (Å, °)

**Table:**

*D*—H···*A*	*D*—H	H···*A*	*D*···*A*	*D*—H···*A*
N2—H2A···O1^ii^	0.86	1.95	2.787 (3)	165

Symmetry codes: (ii) −*x*+2, −*y*+1, −*z*+1.
